# Endogenous CCN5 Participates in Angiotensin II/TGF-β_1_ Networking of Cardiac Fibrosis in High Angiotensin II-Induced Hypertensive Heart Failure

**DOI:** 10.3389/fphar.2020.01235

**Published:** 2020-09-03

**Authors:** Anan Huang, Huihui Li, Chao Zeng, Wanli Chen, Liping Wei, Yue Liu, Xin Qi

**Affiliations:** ^1^ Nankai University School of Medicine, Tianjin, China; ^2^ Department of Cardiology, Tianjin Union Medical Center, Tianjin, China; ^3^ Graduate School, Tianjin University of Traditional Chinese Medicine, Tianjin, China

**Keywords:** angiotensin II, CCN5, cardiac fibrosis, heart failure, hypertension

## Abstract

Aberrant activation of angiotensin II (Ang II) accelerates hypertensive heart failure (HF); this has drawn worldwide attention. The complex Ang II/transforming growth factor (TGF)-β_1_ networking consists of central mechanisms underlying pro-fibrotic effects; however, this networking still remains unclear. Cellular communication network 5 (CCN5), known as secreted matricellular protein, mediates anti-fibrotic activity by inhibiting fibroblast-to-myofibroblast transition and the TGF-β_1_ signaling pathway. We hypothesized that endogenous CCN5 plays an essential role in TGF-β_1_/Ang II networking-induced cardiac fibrosis (CF), which accelerates the development of hypertensive HF. This study aimed to investigate the potential role of CCN5 in TGF-β_1_/Ang II networking-induced CF. Our clinical retrospective study demonstrated that serum CCN5 decreased in hypertensive patients, but significantly increased in hypertensive patients taking oral angiotensin-converting enzyme inhibitor (ACEI). A negative association was observed between CCN5 and Ang II in grade 2and 3 hypertensive patients receiving ACEI treatment. We further created an experimental model of high Ang II-induced hypertensive HF. CCN5 was downregulated in the spontaneously hypertensive rats (SHRs) and increased *via* the inhibition of Ang II production by ACEI. This CCN5 downregulation may activate the TGF-β_1_ signaling pathway, which promotes direct deposition of the extracellular matrix (ECM) and fibroblast-to-myofibroblast transition *via* activated Smad-3. Double immunofluorescence staining of CCN5 and cell markers of cardiac tissue cell types suggested that CCN5 was mainly expressed in the cardiac fibroblasts. Isolated cardiac fibroblasts were exposed to Ang II and transfected with small interfering RNA targeting CCN5. The expression of TGF-β_1_ together with Col Ia and Col IIIa was further promoted, and alpha-smooth muscle actin (α-SMA) was strongly expressed in the cardiac fibroblasts stimulated with Ang II and siRNA. In our study, we confirmed the anti-fibrotic ability of endogenous CCN5 in high Ang II-induced hypertensive HF. Elevated Ang II levels may decrease CCN5 expression, which subsequently activates TGF-β_1_ and finally promotes the direct deposition of the ECM and fibroblast-to-myofibroblast transition *via* Smad-3 activation. CCN5 may serve as a potential biomarker for estimating CF in hypertensive patients. A novel therapeutic target should be developed for stimulating endogenous CCN5 production.

## Introduction

Cardiovascular disease is the leading cause of deaths, accounting for 17.7 million deaths of 55 million deaths worldwide in 2017 (Yasin et al., 2019; Yasin et al., 2019). Hypertension is the main risk factor for cardiovascular disease and may lead to increased morbidity of coronary artery disease, heart failure (HF), and myocardial infarction. The worldwide prevalence of hypertension and the associated complication, especially HF secondary to hypertension, have drawn attention (Dagenais et al., 2019). Long-term high blood pressure (BP) may promote the development of pathological cardiac structural and functional deterioration, leading to left ventricular (LV) hypertrophy and cardiac fibrosis (CF). These irreversible cardiac remodeling responses always culminate into HF eventually (Lai et al., 2019).

Over-activation of the renin-angiotensin-aldosterone system (RAAS), as the major cause of juvenile hypertension, is often characterized by aberrant activation of angiotensin II (Ang II). Over-expression of Ang II affects regulation of high BP and CF, eventually leading to HF (Berk et al., 2007; Singh and Karnik, 2019). In this high Ang II-induced hypertensive HF, Ang II type 1 receptor, bound to Ang II, may activate transforming growth factor-β_1_ (TGF-β_1_), which subsequently promotes deposition of the extracellular matrix (ECM) and sensitize fibroblast-to-myofibroblast transition (Nagpal et al., 2016). Downregulation of Ang II expression by blocking the conversion of angiotensin I (Ang I) to Ang II using an angiotensin-converting enzyme inhibitor (ACEI) prevents cardiac function deterioration from HF in hypertensive patients (Zhang et al., 2019). In clinical practice, ACEI has high recommendation level in treatment of high Ang II-induced hypertensive HF (Yancy et al., 2017).

The cellular communication network (CCN) family, known as a group of matricellular proteins, has been described with variant cell functions in regulating fibrosis, angiogenesis, cell differentiation, and wound repair (Xu et al., 2015; Jeong et al., 2016). Several members of the CCN family play essential roles in the development of pressure overload-induced myocardial fibrosis. CCN2 (cellular communication network 2), also called as connective tissue growth factor, is a pro-fibrotic mediator in the development of CF, which can be induced by TGF-β_1_ in cardiac fibroblasts and cardiomyocytes (Ye et al., 2019). Besides these pro-fibrotic effects of CCN2, the anti-fibrotic potential of cellular communication network 5 (CCN5, Wisp-2) (Grunberg et al., 2018). As secreted proteins, CCN2 and CCN5 play opposing roles in the development of CF. The possible mechanisms underlying anti-fibrotic effects involve in blocking fibroblast-to-myofibroblast transition, endothelial-mesenchymal transition, and the TGF-β_1_ signaling pathway (Jeong et al., 2016). Although several studies have reported the anti-fibrotic effects of exogenous CCN5 in HF, the roles of endogenous CCN5 in high Ang II-induced hypertensive HF still remain unclear (Yoon et al., 2010; Jeong et al., 2016). We hypothesized that endogenous CCN5 plays an essential role in TGF-β_1_/Ang II networking-induced CF which accelerates the development of hypertensive HF. We aimed to investigate the potential role of CCN5 in TGF-β_1_/Ang II networking-induced CF.

## Methods and Materials

For expanded and detailed information about the human study population, reagents, BP measurement, echocardiography, histopathology, ELISA estimation, reverse transcription and real-time quantitative polymerase chain reaction, protein extraction, Western blotting, neonatal rat cardiomyocytes, and cardiac fibroblasts culture, small interfering RNA transfection, and immunofluorescence assay, please refer to the **Supplementary Materials**.

### Human Study Population

All protocols were approved by the Ethical Committee Board of Tianjin Union Medical Center, and all subjects provided informed consent.

### Animals Model of Hypertensive Heart Failure

Spontaneously hypertensive rats (SHRs) obtained by selective inbreeding of the Wistar-Kyoto rats (WKYs; Vital River, Beijing, China) with a genetic basis for high BP were selected for mimicking hypertension. Normotensive WKY (Vital River, Beijing, China) were chosen as the negative controls. Twenty-four 13-week-old SHRs (weight 200 ± 20 g) were equally divided into the model group or enalapril group based on whether enalapril treatment [SFDA approval number H20170298, 5 mg/tablet, Merck Sharp & Dohme (Australia) Pty. Ltd] was given or not. Additionally, the control group comprised 13-week-old WKYs (n = 12). The animals were housed in a 12-h light/dark room and given free access to tap water and chow feed under laboratory conditions. This experiment proceeded after acclimatization in an on-site facility for 1 week. The enalapril group animals were administered with enalapril [1.05 mg. (Kg. day)^−1^] orally in a 1 ml of distilled water; accordingly, animals in the control and model groups were administered with 1 ml of distilled water using a disposable plastic syringe.

### Statistical Analysis

Data were analyzed using SPSS version 17.0 (SPSS Inc., Chicago, USA). Discrete variables were expressed as numbers and percentages. Mean ± SD or median with interquartile (IQ) 25% and 75% (Q25–Q75) were based on the normality for continuous variables. Normality for continuous variables was performed by the Kolmogorov-Smirnov tests. Categorical variables were analyzed using chi-square tests. To compare groups, we used the Mann-Whitney U-test followed by Tukey’s multiple comparison tests or Kruskal-Wallis tests to analyze non-normally distributed continuous variables. Spearman correlation analysis was performed for non-conformity analysis. In all analyses, statistical significance was accepted at *P* < 0.05.

## Results

### Demographic Characteristics

A total of 380 hypertensive patients and 39 normotensive subjects (control) were enrolled into this study. All characteristics of hypertensive patients across BP categories [grade 1 (n = 50), mean BP 149.76/87.84 mmHg; grade 2 (n = 110), mean BP 163.69/94.22 mmHg; grade 3 (n = 220), mean BP 190.21/104.03 mmHg] were demonstrated. There were no differences in age, nor history of hypertension between the hypertensive patients and the normotensive subjects (*P* > 0.05) (**Supplemental Table 1**). Compared to normotensive subjects, the heart rate levels of all hypertensive patients increased significantly (*P* < 0.05). The distribution of sex, drinking, and smoking was comparable between the hypertensive patients and normotensive subjects (*P* > 0.05). Additionally, no statistical difference was demonstrated in the lipid metabolism and renal function between hypertension and control groups (*P* > 0.05). Although the median body mass index (BMI) increased gradually with hypertension grade in patients, no significant difference was demonstrated among diverse sub-groups (*P* > 0.05). Cardiac structure deterioration was observed in the hypertensive patients, especially in patients with grade 3 hypertension [left atrium (LA), LV, left ventricle posterior wall (LVPW), interventricular septum (IVS), *P* < 0.001]. Cardiac systolic function [left ventricular ejection fraction (LVEF): *P* > 0.05] showed no difference among diverse sub-groups. However, the E/A ratio, an echocardiographic index for evaluating diastolic dysfunction, decreased significantly with increasing hypertension grade (*P* < 0.05). To indirectly evaluate the extension of CF, we calculated the left ventricular mass index (LVMI) value for each subject. The mean LVMI level were significantly increased with increasing hypertension grade (*P* < 0.01).

### Elevation of Serum Ang II, CCN2, and CCN5

**Figure 1:**
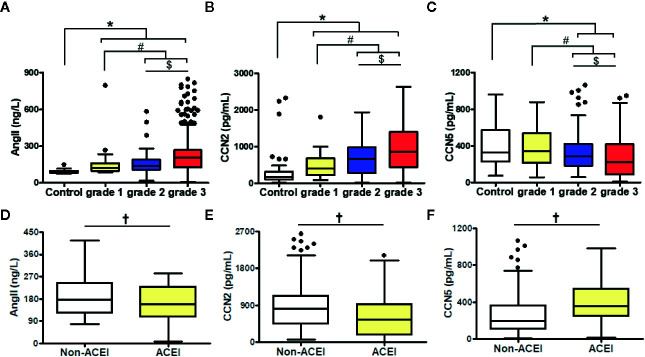
Comparison of Ang II **(A, D)**, CCN2 **(B, E)**, and CCN5 **(C, F)** concentrations in different of blood pressure levels among hypertensive patients with or without ACEI. Data were expressed as median with interquartile range (IQR); whiskers represent ± 1.5 IQR (boxplots). ^*^ *P* < 0.05 *vs*. control, ^#^ *P* < 0.05 *vs*. grade 1, ^$^ *P* < 0.05 *vs*. grade 2. **^†^***P* < 0.05 *vs*. No ACEI. grade 1/2/3 were defined as the grade of blood pressure in hypertensive patients. Ang II, angiotensin II; CCN2, cellular communication network factor 2; CCN5, cellular communication network 5; ACEI, angiotensin converting enzyme inhibitor.

Serum Ang II, CCN2 concentrations were higher in the hypertensive patients, compared to normotensive subjects. grade 3 hypertension patients (CCN2: median 855.73 pg/ml, Ang II: median 209.72 ng/L) showed the highest CCN2 and Ang II concentrations compared with grade 1 (CCN2: median 404.13 pg/ml, Ang II: median 122.44 ng/L) and grade 2 patients (CCN2: median 653.43 pg/ml, Ang II: median 141.41 ng/L) (*P* < 0.01) (**Figures 1A, B** **)**.

Contrarily, CCN5 levels decreased along with the increased BP (grade 1: median 344.17 pg/ml, grade 2: median 284.45 pg/ml, grade 3: median 224.01 pg/ml, *P* < 0.05) (**Figure 1C**). ACEI, which inhibits the conversion of Ang I to Ang II, could attenuate the expression of Ang II. CCN5 can be secreted into circulating blood from multiple vital organs including the heart, lung, and adipose tissue. To evaluate the effects of lung and adipose on serum CCN5 levels, the expression of CCN5 in the lung and adipose tissue was determined *via* Western blotting assay. No differences were showed in the expression of CCN5 between WKY and SHR in the lung and adipose tissue. However, the expression of CCN5 in adipose tissue was higher than that in the lung (WKY: *P* < 0.05, SHR: *P* < 0.05). This suggested that the change in serum CCN5 levels mainly be affected by production and secretions of the heart in normotensive and hypertensive subjects (**Figure S1**).

Moreover, we explored whether the downregulation of Ang II could affect the serum CCN2 or CCN5 levels in hypertensive patients (**Figures 1D–F**). We cataloged all hypertensive patients across ACEI usage rates. Serum Ang II levels were decreased after ACEI treatment in hypertensive patients (*P* < 0.05). Furthermore, we found that hypertensive patients using ACEI had elevated CCN2 and lower CCN5 levels (*P* < 0.05).

### Association Between Ang II and CCN2/CCN5

**Figure 2:**
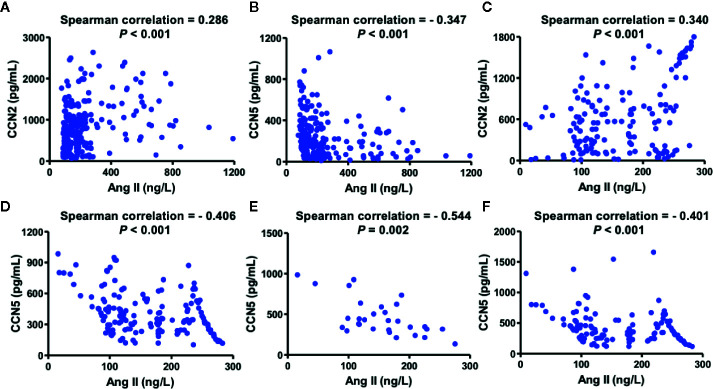
Association between serum CCN2/CCN5 and serum Ang II in hypertensive patients. **(A)** Spearman analysis between serum CCN2 and serum Ang II in hypertensive patients without ACEI. **(B)** Spearman analysis between serum CCN5 and serum Ang II in hypertensive patients without ACEI. **(C)** Spearman analysis between serum CCN2 and serum Ang II in hypertensive patients with ACEI. **(D)** Spearman analysis between serum CCN5 and serum Ang II in hypertensive patients with ACEI. **(E)** Spearman analysis between serum CCN2 and serum Ang II in patients with grade 2 hypertension on treatment with ACEI treatment. **(F)** Spearman analysis between serum CCN2 and serum Ang II in patients with grade 3 hypertension on ACEI treatment. CCN2, cellular communication network factor 2; CCN5, cellular communication network 5; Ang II, angiotensin II; ACEI, angiotensin converting enzyme inhibitor.

Spearman analysis was performed to evaluate whether serum CCN2 and CCN5 are related to serum Ang II. To investigate whether downregulating Ang II had an influence on this association, all hypertensive patients were divided into two sub-groups according to the usage of ACEI (**Figures 2A–D**). Serum CCN2 levels (r = 0.286, *P* < 0.01) and serum CCN5 levels (r = −0.347, *P* <0.01) correlated with serum Ang II levels in hypertensive patients without ACEI treatment. Coincidently, these Spearman rank relationships were further enhanced in hypertensive patients using ACEI (CCN2: r = 0.340, *P* < 0.01; CCN5: r = −0.406, *P* < 0.01). Afterward, we investigated this association between Ang II and CCN5 further from grade 1 to grade 3 hypertensive patients with ACEI or not. These results demonstrated that negative association between Ang II and CCN5 was found in grade 2, and 3 hypertensive patients with the treatment of ACEI respectively (grade 2: r = −0.544, *P* < 0.01; grade 3: r = −0.401, *P* < 0.001) (**Figures 2E, F** **)**.

### Characterization of High Ang II-Induced Hypertensive Heart Failure

**Figure 3:**
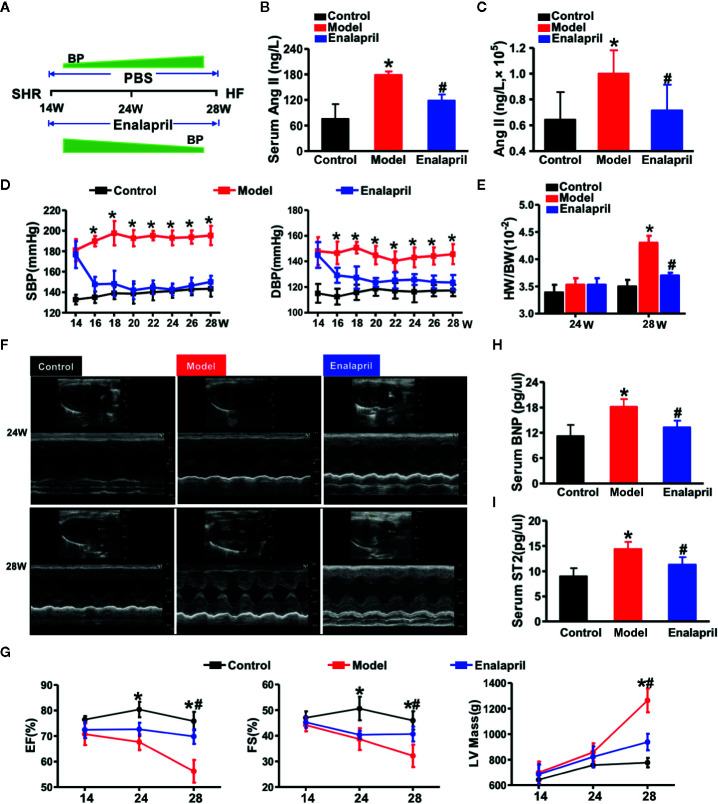
High Ang II-induced hypertensive heart failure. **(A)** Illustration of the experimental strategy in this work. **(B, C)** Ang II concentrations in serum or myocardial tissue. *P < 0.05 vs. control, ^#^ *P* < 0.05 vs. model. **(D)** Blood pressure reduction in SHRs treated by enalapril for 14 weeks. *P < 0.05 vs. model. **(E)** Ratio of heart weight and body weight in SHR or treated by enalapril at 28 weeks. *P < 0.05 vs. control, ^#^ *P* < 0.05 vs. model. **(F)** Echocardiographic phenotype in SHRs or treated by enalapril at 24 and 28 weeks respectively. **(G)** Quantification of cardiac function at different time-points (EF, FS, LV mass). ^*^ *P* < 0.05 vs. control, ^#^ *P* < 0.05 vs. model. **(H, I)** Serum biomarker concentrations of myocardial injuries (BNP, and sST2) in SHRs at 28 weeks. ^*^ *P* < 0.05 vs. control, ^#^ *P* < 0.05 vs. model. Ang II, angiotensin II; SHRs, spontaneously hypertensive rats; EF, ejection fraction; FS, fractional shortening; LV mass, left ventricular mass; BNP, brain natriuretic peptide; sST2, soluble suppression of tumorigenicity-2.

After 14 weeks, the SHR model group exhibited a higher expression of Ang II in both serum and myocardial tissue than the WKYs of the control group. Moreover, the Ang II expression in the serum and myocardial tissue could be downregulated using enalapril (**Figures 3A–C**). Over the 14-week observation period, both SBP and DBP of the model group (SHR) were increased markedly compared to those of the control group (WKY) (**Figure 3D**). Enalapril decreased systolic blood pressure (SBP) and diastolic blood pressure (DBP) levels immediately. There was no difference in the ratio of the heart and body weight between the model and control groups until week 28 (**Figure 3E**).

Echocardiography was performed at distinct time points of 24- and 28-week to determine the success of the experimental model of hypertensive HF (**Figures 3F, G** **)**. LV mass, as one of the essential cardiac hypertrophic indices, was increased in the model group with decreased LVEF and fraction shortening (FS) value at 24 weeks. The cardioprotective effects of enalapril from inhibiting Ang II, was not apparent until week 28. Enalapril protected LVEF, FS, and LV mass from deterioration. Enarapril could ameliorate cardiac dysfunction in the high Ang II-induced HF, but this protective effect depended on the persistent inhibition of Ang II. Subsequently, serum BNP and sST2 (soluble suppression of tumorigenicity-2) levels were detected, indicating that enalapril could attenuate high Ang II-induced hypertensive HF (**Figures 3H, I** **)**.

### Endogenous CCN5 and Ang II-TGF-β_1_ Signaling Axis Networking in Hypertensive Heart Failure

**Figure 4:**
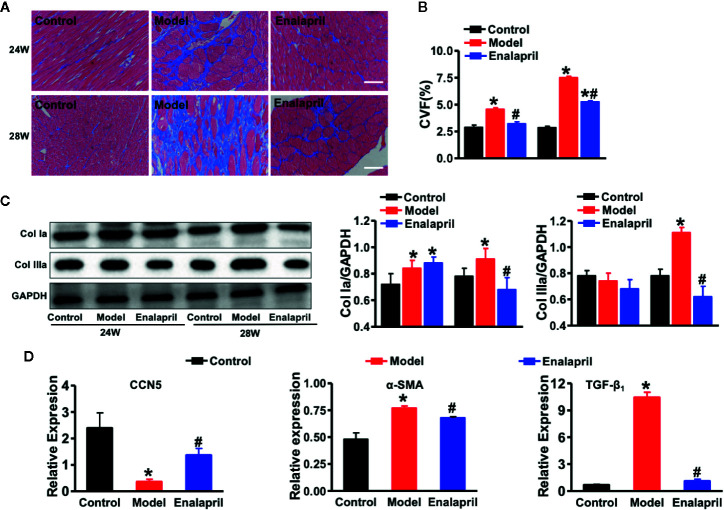
Anti-fibrotic effects of CCN5 in high Ang II-induced hypertensive heart failure at 28 weeks. **(A)** Representative Masson staining of myocardial tissue from different groups at 24 and 28 weeks. Scale bar 100 μm. **(B)** Quantification of CVF from different groups at 24 and 28 weeks. ^*^ *P* < 0.05 vs. control, ^#^ *P* < 0.05 vs. model. **(C)** Western blot analysis of Col Ia and Col IIIa from different groups at 24 and 28 weeks. Quantification of Col Ia and Col IIIa in SHRs or treated by enalapril at 24 and 28 weeks. *P < 0.05 vs. control, ^#^ *P* < 0.05 vs. model. **(D)** Relative expression of CCN5, α-SMA, and TGF-β1 in SHRs or treated by enalapril at 24 and 28 weeks. ^*^ *P* < 0.05 vs. control, ^#^ *P* < 0.05 vs. model.

After evaluation of cardiac structure, and function, our results indicated that long-term stimuli of high BP could induce CF, hypertrophy, and even severe HF. We evaluated the morphological changes of the heart tissue further. Myocyte hypertrophy occurred significantly in the model group from week 24; however, this kind of hypertrophy was reversed by enalapril at week 28 (**Figure S2**). Myocardial fibrosis participated in the entire cardiac hypertrophy process. Collagen deposition occurred in the model group at week 24 and further worsened at week 28. Enalapril could effectively attenuate this collagen deposition in SHRs at 24 and 28 weeks (**Figures 4A, B** **)**. These results coincided with the expression of collagen Ia and collagen IIIa (**Figure 4C**).

**Figure 5:**
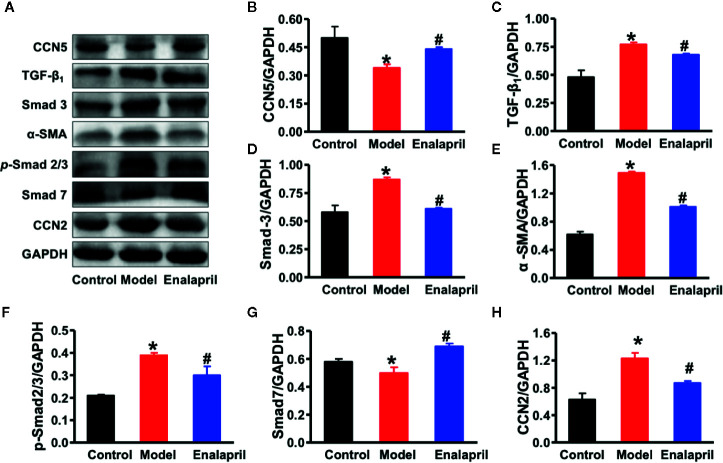
**(A)** Representative Western blot analysis of CCN5, TGF-β_1_, Smad-2, Smad-3, α-SMA, p-Smad-2/3, Smad-7, and CCN2 in myocardial tissue among different groups. **(B–H)** Quantification of CCN5, TGF-β_1_, Smad-2, Smad-3, α-SMA, p-Smad-2/3, Smad-7, and CCN2 expression in myocardial tissue among different groups. ^*^ *P* < 0.05 vs. control, ^#^ *P* < 0.05 vs. model. CVF, collagen volume fraction; Col Ia, collagen Ia; Col IIIa, collagen IIIa; SHRs, spontaneously hypertensive rats; CCN5, cellular communication network 5; α-SMA, alpha smooth muscle actin; TGF-β1, transforming growth factor-β1; CCN2, cellular communication network 2.

A previous study demonstrated that CCN5 could block the TGF-β_1_ signaling pathway and fibroblast-to-myofibroblast transition (Jeong et al., 2016). In our study, we found that TGF-β_1_ and alpha-smooth muscle actin (α-SMA) were also elevated, which indicated that pro-fibrotic pathways and fibroblast-to-myofibroblast transition were activated in the model group (**Figures 4D**). These results demonstrated that endogenous CCN5 might create a link between Ang II and TGF-β_1_ and α-SMA. Thereafter, we investigated the interaction of CCN5 and Ang II-TGF-β_1_ signaling axis in fibrotic pathways. The expression of myocardial CCN5 was significantly reduced in the model group and in reverse increased after inhibition of Ang II by enalapril (**Figure 4D**). The results of Western blotting analysis also revealed decreased endogenous CCN5 and an activated TGF-β_1_ signaling pathway and fibroblast-to-myofibroblast transition (**Figures 5A–H**).

**Figure 6:**
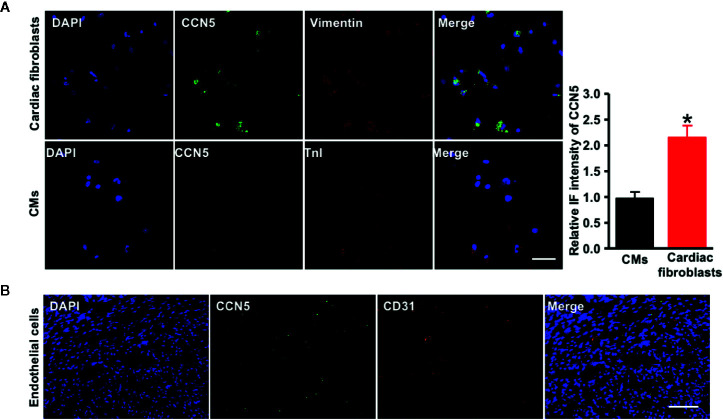
CCN5 expression in major rat cardiac cell types. **(A)** Double immunofluorescence-staining of CCN5 and cell markers (vimentin/TnI) in CFs and CMs. Scale bar 30 μm. Quantitative analysis of intensive immunofluorescence density of CCN5. ^*^ *P* < 0.05 vs. CMs. **(B)** Double immunofluorescence-staining of CCN5 and CD31 (cell marker of cardiac ECs), scale bar 100 μm. CCN5, cellular communication network 5; CFs, cardiofibroblasts; CMs, cardiomyocytes; ECs, endothelial cells; IF, immunofluorescence.

To confirm the main resource of CCN5 in cardiac tissues, we isolated the rat neonatal cardiomyocytes (CMs) and cardiac fibroblasts from neonatal WKY. Then we performed double immunofluorescence staining of CCN5 and cell markers (TnI or vimentin) of CMs and cardiac fibroblasts. CCN5 was mainly expressed in cardiac fibroblasts (*P* < 0.05 *vs*. CMs) (**Figure 6A**). Additionally, we performed the double immunofluorescence staining of CCN5 and CD31 (cell marker of cardiac endothelial cells). No clear co-localization was found between CCN5 and CD31 in cardiac tissues, and this demonstrated that CCN5 may not be mainly expressed in cardiac endothelial cells (**Figure 6B**).

**Figure 7:**
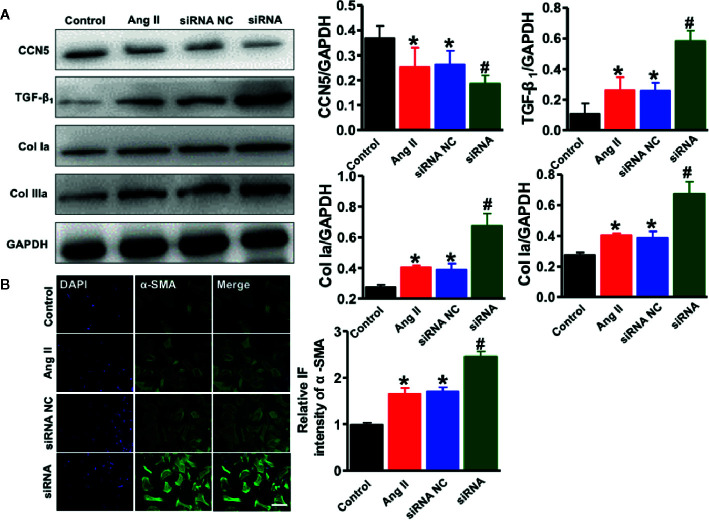
Downregulation of CCN5 enhanced Ang II-induced activation of pro-fibrotic effects. **(A)** Representative Western blot analysis of CCN5, TGF- β_1_, Col 1a, and Col IIIa. ^*^ *P* < 0.05 vs. control, ^#^ *P* < 0.05 vs. Ang II/siRNA NC. ^*^ *P* < 0.05 vs. control, ^#^ *P* < 0.05 vs. Ang II/siRNA NC. CCN, cellular communication network 5; TGF, transforming growth factor. **(B)** Representative image of immunofluorescent staining of myofibroblasts markers (α-SMA, Scale bar 50 μm. *P < 0.05 vs control, ^#^ *P* < 0.05 vs Ang II/siRNA NC.

To evaluate the essential role of endogenous CCN5 in the Ang II induced profibrotic pathophysiology, the isolated cardiac fibroblasts were exposed to Ang II (0.1 μM). The siRNA targeting CCN5 was synthesized and transfected into cardiac fibroblasts to suppress the CCN5 expression. After stimulation with Ang II, CCN5 expression was significantly down-regulated in the cardiac fibroblasts, and this downregulation was further enhanced after on using siRNA (^*^ *P* < 0.05 *vs*. control; ^#^ *P* < 0.05 *vs*. Ang II/scrambled siRNA) (**Figure 7A**). The expression of TGF-β_1_, Col Ia, and Col IIIa was also upregulated on use of siRNA. These results confirmed that Ang II promoted the TGF-β_1_ induced CF by down-regulating the expression of CCN5. We investigated the effects of downregulation of CCN5 on fibroblast-to-myofibroblast transition. siRNA could significantly promote expression of α-SMA in the cardiac fibroblasts, suggesting that fibroblast-to-myofibroblast transition was enhanced by downregulation of CCN5 expression (**Figure 7B**).

## Discussion

In this study, we demonstrated that CCN5 downregulation might be closely related to Ang II expression in hypertensive HF. CCN5 expression could be elevated by inhibiting Ang II, which provided a cardioprotective effect in hypertension-induced HF. Serum CCN5, CCN2, and Ang II concentrations were tested between hypertensive patients and healthy controls, and we further investigated on the association between Ang II and matricellular proteins of CCN5 and CCN2. Using our experimental model of high Ang II-induced hypertensive HF along with elevated expression of Ang II in both serum and myocardial tissue, we evaluated whether downregulation of CCN5 could affect the cardiac structure, function, and myocardial fibrosis.

Moreover, we elucidated the indispensable role of endogenous CCN5 in high Ang II-induced hypertensive HF. Our clinical results demonstrated that serum CCN5 levels reduced significantly because of the increased severity and history of high BP in hypertensive patients. Additionally, this negative association was described between serum Ang II and CCN5, especially in grade 2 and 3 hypertensive patients using oral ACEI regularly. Our rat model of essential hypertensive HF revealed a significant decrease of CCN5 in high Ang II-induced hypertensive HF. Expression of CCN5 was upregulated after ACEI treatment, which further reversed myocardial fibrosis and protected heart function *via* inhibition of TGF-β_1_ signaling and fibroblast-to-myofibroblast transition.

CCN5 has multiple biological functions (Bornstein and Sage, 2002). Unlike the other CCN family proteins, CCN5 specially lacks a cysteine-rich carboxyl-terminal repeat domain, suggesting that it may be an alternative regulator of other CCN family proteins. Comparison studies of CCN2 with prominent pro-fibrotic activity in cardiac remodeling, in which CCN5 is best characterized, reported anti-hypertrophic and anti-fibrotic effects in the heart (Jeong et al., 2016; Ye et al., 2019). Besides cardiac tissue, lung and adipose tissue show high CCN5 expression (Hammarstedt et al., 2013; Fiaturi et al., 2018; Grunberg et al., 2018). To exclude interference of serum CCN5 from secreted CCN5 from lung and adipose tissue, we compared the expression of CCN5 in lung and adipose tissue between WKY and SHR. The results suggested that CCN5 expression in lung and adipose tissue might not cause the differentiated expression of CCN5 in the serum. Therefore, we assessed the serum CCN5 levels to predict the expression of CCN5 in cardiac tissue. To the best of our knowledge, these protective effects of CCN5 in the heart were verified *via* supplementation of exogenous CCN5. However, whether expression of endogenous CCN5 could be modulated by exogenous stimuli and play an indispensable role in anti-hypertrophic and anti-fibrotic activity remained unclear.

Ang II is known as the primary regulatory factor in a series of RAAS-induced physiological and pathophysiological actions, which participates in homeostatic control of arterial pressure, tissue perfusion, and extracellular volume (Park et al., 2019). Active Ang II is converted from Ang I by ACE, which is cleaved by renin. High Ang II expression induced by RAAS over-activation contributes to the pathophysiology of diseases such as hypertension and hypertensive HF (Rosenkranz, 2004). Our results demonstrated that Ang II increased gradually in hypertensive patients with increasing BP levels. Additionally, we created an experimental model of hypertensive HF with increased Ang II in the serum and myocardial tissue. These results implied that our experimental model mimicked a high Ang II-induced hypertensive HF.

To verify the essential role of CCN5 in hypertension, firstly we detected decreased CCN5 levels, but elevated CCN2 levels in all hypertensive patients. A previous study indicates opposite effects of CCN2 and CCN5 on the regulation of CF, which is consistent with our results (Jeong et al., 2016). Through the comparison of CCN5 levels in hypertensive patients with and without ACEI treatment and further associated analysis, we found that patients with higher Ang II levels had lower concentrations of CCN5, which suggested that an interaction might exist between Ang II and CCN5. In our experimental model, we found that CCN5 was mainly expressed in cardiac fibroblasts, but not cardiomyocytes or cardiac endothelial cells. High Ang II expression could downregulate CCN5 expression to promote CF and deteriorate cardiac systolic and diastolic functions. Meanwhile, Ang II can be attenuated using ACEI, followed by ameliorateion of myocardial fibrosis and cardiac function. During this process, we detected activated TGF-β_1_, which both promoted direct deposition of ECM, and fibroblast-to-myofibroblast transition *via* activated Smad-3 (Nagpal et al., 2016).

Fibroblasts within a healthy working heart control the secretion and maintenance of ECM components and more importantly regulate the transmission of mechanical and electrical stimuli (Santiago et al., 2010). In hypertensive-induced cardiac hypertrophy and fibrosis, the phenotype conversion of cardiac fibroblast-to-myofibroblast is a critical event, this could precipitate HF (Czubryt, 2019). With elevation of BP, cardiac fibroblasts become overactivated and converted to myofibroblasts in response to pressure overload. During this process, several key phenotypic markers of myofibroblast are recognized including α-SMA and periostin (Bagchi et al., 2016). In this study, high α-SMA expression was found in the myocardial tissue of SHRs, opposed to the decreased expression after downregulation of Ang II using ACEI. After inhibiting the expression of CCN5 in the cardiac fibroblasts, we found a significant increase of α-SMA in the cardiac fibroblasts. These results demonstrated that Ang II might promote the phenotype conversion of cardiac fibroblast-to-myofibroblast by directly inhibiting the expression of CCN5.

Although the fibroblast-to-myofibroblast conversion has been described previously, the signaling mechanisms governing this conversion were not yet clearly elucidated. Phenotype fibroblast-to-myofibroblast conversion can be induced by mechanical tension, or TGF-β_1_ stimuli, which aids in quick ECM pathological remodeling (Roche et al., 2015). The TGF-β_1_-Smad signaling pathway, which is arguably one of the most potent inductive mechanisms, is involved in this process (Roche et al., 2015). Obviously, healthy myocardial tissue was devoid of myofibroblasts, but myofibroblasts became abundant after receiving several stimulating factors, which promoted hypersecretion of ECM components such as collagen type I, periostin, and fibronectin (Tomasek et al., 2002). Excessive ECM components produced by myofibroblast accelerate CF and even HF.

Taken together, the results of this study showed that serum CCN5 was reduced significantly in hypertensive patients and increased in hypertensive patients using ACEI. The negative association between CCN5 and Ang II in the serum indicated that Ang II interacted with CCN5. Our experimental model of high Ang II-induced hypertensive HF revealed that CCN5 was downregulated in the high Ang II SHR and increased *via* Ang II production inhibition by ACEI treatment. Thereafter, this downregulation of CCN5 activates TGF-β_1_, which promotes direct deposition of ECM, and fibroblast-to-myofibroblast transition *via* activated Smad-3.

The current study highlights the essential role of endogenous CCN5 in CF. CCN5 participates in the Ang II/TGF-β_1_ networking. However, this networking is a vastly and complicated process, endogenous CCN5 may interact with multiple signaling factors including matrix metalloproteases and metalloproteases within the Ang II/TGF-β_1_ networking (Jeong et al., 2016). This study cannot cover all of biological functions of endogenous CCN5 within this networking. Further work will focus on the crucial role of endogenous CCN5 in degradation of the ECM.

In summary, we verifiy the essential role of endogenous CCN5 in high Ang II-induced hypertensive HF. Elevated Ang II inhibit CCN5 expression, which subsequently activates TGF-β_1_ and finally promotes direct deposition of ECM and fibroblast-to-myofibroblast transition *via* Smad-3 activation. CCN5 can be used as a potential biomarker for estimating CF in hypertensive patients. A novel therapeutic target can be developed for stimulating endogenous CCN5 production.

## Data Availability Statement

The raw data supporting the conclusions of this article will be made available by the authors, without undue reservation, to any qualified researcher.

## Ethics Statement

The studies involving human participants were reviewed and approved by Tianjin Union Medical Center. The patients/participants provided their written informed consent to participate in this study. The animal study was reviewed and approved by Tianjin Union Medical Center.

## Author Contributions

AH designed and completed the experiments, analyzed the data, and drafted the manuscript. HL analyzed the data, collected the clinical data, and analyzed the clinical data. CZ and WC completed the experiments and collected the clinical data. LW revised the draft. XQ conceived this study and finalized the manuscript. All authors contributed to the article and approved the submitted version.

## Funding

The study was supported by the major projects of Science and Technology Committee of Tianjin (grant number 16ZXMJSY00060); the Tianjin Health Bureau Key Project Fund (grant number 16KG155); and the Science and Technology Project of Tianjin Union Medical Center (grant number 2019YJZD001).

## Conflict of Interest

The authors declare that the research was conducted in the absence of any commercial or financial relationships that could be construed as a potential conflict of interest.

## Abbreviations

Ang II, angiotensin II; HF, heart failure; TGF, transforming growth factor; CCN5, cellular communication network 5; ACEI, angiotensin-converting enzyme inhibitor; SHR, spontaneously hypertensive rats; CCN2, cellular communication network 2; ECM, extracellular matrix; LV, left ventricular; RAAS, renin-angiotensin-aldosterone system; Ang I, angiotensin I; CF, cardiac fibrosis; WKY, Wistar-Kyoto rat; LVPW, left ventricle posterior wall; IVS, interventricular septum; LVEF, left ventricular ejection fraction; FS, fraction shortening; α-SMA, alpha-smooth muscle actin.
