# Supplementary material for: Endogenous CCN5 Participates in Angiotensin II/TGF-β1 Networking of Cardiac Fibrosis in High Angiotensin II-Induced Hypertensive Heart Failure
Source: Front Pharmacol. 2020 Sep 3;11:1235. doi: 10.3389/fphar.2020.01235 (PMC7494905; doi:10.3389/fphar.2020.01235)
Supplement: Supplementary file 1 [file DataSheet_1.docx]

**Endogenous CCN5 participates in angiotensin II/TGF-β_1_ networking of cardiac fibrosis in high Angiotensin II induced hypertensive heart failure**

Anan Huang^1,2^, Huihui Li^3^, Chao Zeng^2^, Wanli Chen^3^, Liping Wei^2^, Yue Liu^2^, Xin Qi^1,2^^*^

1. Nankai University School of Medicine, Tianjin, 300071, China 2. Department of Cardiology, Tianjin Union Medical Center, 300121, China 3. Graduate School, Tianjin University of Traditional Chinese Medicine, Tianjin, 300193, China

**Correspondence to**: Xin Qi, Department of Cardiology, Tianjin Union Medical Center, 190 Jieyuan Road, Tianjin, P.R. China, 300121, Ph: +86-22-27557112, Fax: +86-22-87721989, E-mail: qixinx2011@yeah.net

**Methods and Materials**

*Human study populations*

A total of 419 consecutive essential hypertensive subjects (217 men and 163 women) were eligible for this retrospective clinical study in the department of cardiology, Tianjin Union Medical Center (Tianjin, PR. China) from September 2016 to August 2018. Hypertension was defined as seated, resting systolic blood pressure (SBP) ≥ 140 mmHg or diastolic blood pressure (DBP) ≥ 90 mmHg at both the screening and recruitment or using any antihypertensive treatment before. All the hypertensive subjects were then split into three sub-groups (Grade 1 [SBP: 140-160mmHg and/or DBP: 90-100mmHg], Grade 2 [SBP: 160-180mmHg and/or DBP: 100-110mmHg], and Grade 3 [SBP: ≥ 160mmHg and/or DBP: ≥ 110mmHg]) according to the grade of blood pressure.

The family history of hypertension was defined as either parent with hypertension. The control group consisted of 39 normotensive blood donors (22 men and 17 women) from physical examination center, Tianjin Union Medical Center (Tianjin, PR. China) with mean age of 65.82 years, who had no symptoms and signs of cardiovascular disease. All the participants underwent laboratory, echocardiographic and physical examination. Exclusion criterial for this study included (1) secondary hypertension, (2) unavailable clinical data, (3) severe renal insufficiency or liver dysfunction, and (4) malignant tumor.

*Reagents*

The reagents in this study were provided from the source indicated. In brief, Enalapril Maleate (Enalapril) was purchased from the Yangtze River Pharm (Yangzhou, Jiangsu Province, China). Angiotensin II (Ang II) was provided from Solarbio (Beijing, China). The bicinchoninic acid assay (BCA) kit and all the quantitative PCR (qPCR) supplies including taqman primer, taqman master mix kits were obtained from Solarbio (Beijing, China). All of qPCR primers were designed and synthesized by Sangon (Shanghai, China). Rat and human specific Ang II, sST2 (soluble suppression of tumorigenicity-2) CCN2, and CCN5 ELISA kits were provided by QIYI Biotech (Shanghai, China). Primary antibodies against CCN2, transforming growth factor-β_1_ (TGF-β_1_), drosophila mothers against decapentaplegic-2 (Smad-2), drosophila mothers against decapentaplegic protein-2 (Smad-3), phosphorylated drosophila mothers against decapentaplegic-2 (p-Smad-2/3), drosophila mothers against decapentaplegic-7 (Smad-7), GAPDH were all purchased from Wanlei Biotech (Shenyang, Liaoning Province, China). Primary antibody against CCN5, and α-SMA and was purchased from ABclonal (Wuhan, Hubei Province, China). Laminin, type II collagenases and ITS Liquid Media Supplement (100 ×) were obtained from Sigma Aldrich (St Louis, MO, USA)*.* Fetal Bovine Serum (FBS) was purchased from Biological Industries (BI) (Beit Haemek, Israel). Dulbecco’s Modified Eagle’s Medium (DMEM), Penicillin-Streptomycin (PS,100 ×) were purchased from Gibco (Gaithersburg, MD, USA). Primary anti-body against Vimentin, TnI, and CD31 was purchased from ABcam (MA, USA). Red- and Green- fluorescence-marked secondary antibody were purchased from Life technology (MA, USA). Small interfering RNA of CCN5 was synthesized by Genepharma Technologies (Jiangsu, China). GP-transfect-Mate reagent was purchased from Genepharma Technologies (Jiangsu, China). HRP-tagged secondary antibody was provided from Zsbio Biology (Beijing, China).

*Measurement of blood pressure*

The rat arterial blood pressure and heart rates of spontaneously hypertensive rats (SHRs) and Wistar-Kyoto rats (WKYs) were measured once a week from 14-week to 28-week. The measurement of blood pressure was reported in previous literature(1). In brief, all the animals were positioned in a 40℃ chamber for appropriately 30 min, the systolic blood pressure (SBP), diastolic blood pressure (DBP) and heart rates were measured using a blood pressure analysis system (CODA-HT8, Kent Scientific Corp, CT, USA).

*Echocardiography*

Rats were anaesthetized mildly with 1.5-3% inhaled isoflurane, and then transthoracic echocardiography was performed to evaluate the cardiac structure and function using an MS-250 ultrasound scanning transducer. The transducer was located to obtain left ventricular (LV) short-axis, long-axis cardiac views using commercially echocardiography system (Vevo 2100, VisualSonics, Toronto, Canada). Left ventricular ejection fraction (LVEF), left ventricular fractional shortening (FS), and LV mass were calculated, and coincidence with the American Society of Echocardiography Guideline(2). Standard echocardiography techniques have been reported previously(3). In brief, cardiac long-axis, or short-axis, M-model tracing of the LV were built at the mid-papillary level, and LV end-systolic, end-diastolic diameter (LVESD, and LVEDD), left ventricle posterior wall (LVPW), and interventricular septum (IVS), left ventricular mass index (LVMI) were measured. Echocardiographic indices were calculated as LVEF (%) = {[7.0/2.4+ LVEDD] × (LVEDD)^3^} × 100%, FS = (LVEDD-LVESD)/LVEDD × 100% and LV mass = {1.04 × (AW+PW+LVEDD)^3^-LVEDD^3^} × 0.8.

*Histopathology*

Rats were sacrificed and then myocardial tissue was fixed in 4% paraformaldehyde (PFA) for 48 h. Fixed tissue was processed routinely and embedded in paraffin. Paraffin sections (5 μm) were cut and mounted on glass slides and stained with Hematoxylin and Eosin (H&E) and Masson’s trichrome staining and examined under a light microscope. The extension of cardiac fibrosis was evaluated by collagen volume fraction (CVF) with the help of ImageJ software. The cross-sectional area (CSA) was performed according to the previous literature(4). Briefly, 50 ovoid cells with visible round nucleus were counted from each LV. Each cell was traced and then the cross-sectional area was determined Images were digitized and processed by Image-Pro Plus software (Media cybernetic, MD, USA).

*ELISA estimation*

The rats were sacrificed and Blood sample or heart tissue was collected. Blood sample was standing at room temperature (RT) for 30 min followed by centrifugation at 3000 rpm for 15 min to obtain the serum. Heart tissue was shredded and homogenized, and then centrifuged at 12,000 rpm for 5 min (4 ℃) to collect supernatant. Brain natriuretic peptide (BNP), soluble suppression of tumorigenicity-2 (sST2) and Ang II concentrations were detected using ELISA assay kits (QIYI Biotech, Shanghai, China). The Ang II concentrations of myocardial tissue was determined by supernatant of heart tissue homogenate. All the procedure was followed manufacturer protocols.

*Reverse transcription (RT) and real-time quantitative polymerase chain reaction*

RNA was isolated from heart tissue of different samples (n = 12) using TRIzol reagent (Sigma-Aldrich, MO, USA) according to manufacturer’s protocol. Total RNA was reverse-transcribed in a 20μl reaction mixture that contained reverse transcriptase, 5×reaction buffer, RNase inhibitor, dNTP mix, and random hexamer primers using the one-step Transcriptor First Strand cDNA Synthesis Kit (Roche, Switzerland). Total RNA concentrations were measured with Biophotometer Plus (Eppendorf, UK), and then equal amounts of total RNA were reverse-transcribed into cDNA using Transcriptor First-Strand cDNA Synthesis (Roche, Switzerland). Real-time RT-PCR was performed on a Step-One Real-Time PCR system (Bio-Rad, USA) using the Fast Start Universal SYBR Green Master Mix Kit (Roche, Switzerland) with specific primers to determine the mRNA expressions of Ang II, α-SMA, and GAPDH. Relative expression of Ang II, and α-SMA among different groups was normalized to the expression of reference gene, GAPDH. The primers of Ang II and GAPDH were provided as following: Ang II (Forward: CAGCATCCTCC-TTGAACTCCA, Reverse: TCGTAGATGGCGAACAGGAA). α-SMA (Forward: CACTTCCTACCCTCGGCAC, Reverse: AGGTGTGGTGC- CAGATCTTC). GAPDH (Forward: GCAAGAGAGAGGCCCTCAG, Reverse: TGTGAGGGAG-ATGCTCAGTG).

*Protein extraction and western blotting*

Protein extraction of myocardial tissue was performed according to the manufacturer’s protocol (KeyGen, Nanjing, China). Quantification of protein was determined using the BCA method (Solarbio, Beijing, China). Protein (30 μg) was resolved in 10% SDS-polyacrylamide gel using TGX stain free kit (Bio-Rad, USA). Protein was transferred into polyvinylidine difluoride (PVDF) membrane (GE Healthcare, USA) after electrophoresis. Blocking of the PVDF membrane was performed using 5% non-fat milk in TBST containing 0.1% tween at RT for appropriately 1 h, followed by primary antibody treatment overnight at 4°C. PVDF membrane was washed with TBST for 5 min (three times). After washing, the PVDF membrane was incubated with the corresponding HRP-labeled secondary antibody at RT for 2h. The PVDF membrane was then washed with TBST (3 times for 5 min each) and finally the blot was visualized using gel doc XR system (Bio-Rad, USA), using ECL reagent (Millipore, USA).

*Neonatal rat cardiomyocytes and cardiac fibroblasts culture*

Primary neonatal rat cardiomyocytes (CMs) and cardiac fibroblasts (CFs) were isolated in line with the method of Simpson(5). Briefly, Ventricles extracted from neonatal WKY rat (<24 h) were enzymatically digested with 0.125% Trypsin at 4℃ for over 12 h, and then transferred into 0.125% collagenase II at 37℃ to collect cell suspension. Cell suspension was cultured on the dishes for over 60 mins to separate cardiac fibroblasts (CFs). Nonattached cells were plated in laminin-coated dishes with DMEM medium with 10% FBS, supplemented with 1% ITS to obtain neonatal CMs.

*Small interfering RNA (siRNA) transfection*

The siRNA (Sense: 5’-GCUCAUGGAACAGUGCUUUTT, Anti-sense: 5’ AAAGCACUGUUCCAUGAGCTT) targeting rat CCN5, and Scrambled siRNA (Sense: 5’-UUCUCCGAACGUGUCACGUTT-3’, Anti-sense: 5’-ACGUGACACGUUCGGAGAATT-3’) was synthesized by Genepharma (Jiangsu, China). For transfection, CFs were cultured to 60-70% confluence and exposed to 100nM CCN5-siRNA and Scrambled siRNA for 6 h using GP-transfect-Mate reagent (Genepharma Technologies) according to the manufacturer’s protocol, and then CFs were cultured to the 10% FBS, 1% PS containing DMEM medium and exposed to 0.1 μM Ang II to induce Ang II induced pro-fibrotic intervene.

Immunoflurescence assay

Cultured Rat CMs and CFs attached to rinsed aseptic coverslips in 24-well plate were washed with PBS for 3 times, and then fixed with 4% PFA for 20 min. 0.1% Triton X-100 was added on the coverslips to permeabilize cells for 30 min, and then cells were blocked with 10% goat serum at RT for 2 h. After incubation with primary antibody against CCN5 and TnI (CMs) / CCN5 and Vimentin (CFs) overnight at 4℃, the cells were then exposed to red- and green- fluorescence-marked secondary antibody at RT for 2 h. Cell nuclei was stained by 4’6-diamidino-2-phenylindole (DAPI) for 10 min. Rat cardiac tissue was collected, fixed in 4% PFA, and 30% sucrose, and then embedded in OCT, frozen in dry ice. Frozen sections were washed with PBS for 3 times to remove the OCT, and then blocked with 5% goat serum for 2 h. Primary antibodies against CD31 and TnI were stained in the cardiac tissue overnight at 4℃. The cardiac tissue was then incubated with red- and green- fluorescence-marked secondary antibodies for 2 h at RT. Cell nuclei was stained by 4’6-diamidino-2-phenylindole (DAPI) for 10 min. Image acquisition was performed on a confocal laser microscope (Olympus, Tokyo, Japan) or Eclipse 80i fluorescence microscope (Nikon).

**Figure S1.** **Representative Western blot analysis of CCN5 in lung and adipose tissues.** ^*^*P* < 0.05 vs Lung. CCN5: Cellular communication network 5. SHRs: Spontaneously hypertensive rats.

**Figure S2. Representative microphotographs of myocytes cross-sectional area. Scale bar 100** μm. ^*^*P* < 0.05 vs control, ^#^*P* < 0.05 vs model.

**Supplemental Table 1. Baseline Characteristics of Hypertensive Patients Compared to Based Controls without hypertension**

| **Variables** | | **Control**  **(n = 39)** | **Grade 1**  **(n = 50)** | **Grade 2**  **(n = 110)** | **Grade 3**  **(n = 220)** | ***P* value** |
| --- | --- | --- | --- | --- | --- | --- |
| **Sex (Male %)**  **Age (years)**  **BMI (Kg/cm^2^)**  **Hypertension**  SBP (mmHg)  DBP (mmHg)  Years  Family history  (n, %)  **HR (beats/min)**  **Smoking (n, %)**  **Drinking (n, %)**  **TC (mmol/L)**  **HDL-c** **(mmol/L)**  **LDL-c (mmol/L)**  **FBG (mmol/L)**  **Cr (mmol/L)**  **ACR (mg/mmol)**  **Echo**  LA (mm)  LV (mm)  LVPW (mm)  IVS (mm)  LVMI (g/m^2^)  E/A  LVEF (%)  **Ang II (ng/L)**  **CCN2 (pg/mL)**  **CCN5 (pg/mL)** | 22 (56.4)  65.82±7.28  23.94 (21.72, 26.64)  119.36±10.92  77.49±8.19  NS  28 (71.8)  66.56±7.09  12 (30.8)  9 (23.1)  4.73 (4.08, 5.15)  1.32 (1.13, 1.61)  3.24 (2.80, 3.52)  5.19 (5.02, 5.65)  69.98±13.04  1.39 (1.04, 2.07)  24 (22, 24)  39 (37, 42)  7.49±1.12  7.49±1.12  59.22±16.24  1.12±0.12  55 (52, 56)  87.82 (81.78, 100.29)  160.58 (98.11, 313.12)  328.01 (228.30, 571.30) | | 34 (68.0)  64.20±10.84  24.52 (22.10, 27.14)  149.76±5.18  87.84±7.99  1 (0, 3.5)  35 (70.0)  74.64±15.24  26 (52.0)  14 (28.0)  4.72 (4.20, 5.35)  1.25 (1.13, 1.45)  3.18 (2.73, 3.81)  5.69 (5.24, 6.22)  66.98±16.66  1.65 (1.09, 3.16)  24 (22, 30)  45 (42, 47)  8.49±0.90  8.5±0.90  98.28±25.44  0.92±0.12  57 (52, 58)  122.44 (99.08, 162.37)  404.13 (220.77, 671.24)  344.17 (212.02, 540.59) | 67 (60.9)  66.79±12.15  24.83 (23.40, 26.44)  163.69±6.36  94.22±10.33  5 (2, 10)  80 (72.7)  77.16±12.56  66 (40.0)  20 (18.2)  4.70 (3.98, 5.34)  1.20 (1.08, 1.41)  2.94 (2.56, 3.51)  5.67 (5.14, 6.64)  71.47±20.94  2.23 (1.10, 4.93)  31 (25, 35)  46 (43, 48)  9.21±1.27  9.23±1.26  98.28±25.44  0.85±0.11  51 (56, 58)  141.41 (110.22, 190.62)  653.43 (269.44, 982.40)  284.45 (183.02, 419.84) | 116 (52.7)  65.52±12.78  25.39 (22.73, 27.85)  190.21±16.67  104.03±14.53  10 (5, 20)  157 (71.4)  74.64±13.03  86 (39.1)  47 (21.3)  4.68 (3.86, 5.08)  1.20 (1.03, 1.40)  3.01 (2.33, 3.55)  6.19 (5.33, 8.16)  76.11±31.16  2.93 (1.28, 6.98)  34 (30, 37)  47 (45, 49)  10.26±1.35  10.35±1.73  121.06±37.13  0.79±0.13  55 (52, 58)  209.72 (129.59, 268.85)  855.73 (440.65, 1398.65)  224.01 (85.68, 418.48) | 0.187  0.607  0.052  <0.001^**^  <0.001^**^  <0.001^**^  0.987  <0.001^**^  0.217  0.600  0.922  0.478  0.701  0.007^**^  0.074  0.077  <0.001^**^  <0.001^**^  <0.001^**^  <0.001^**^  <0.001^**^  <0.001^**^  0.733  <0.001^**^  <0.001^**^  <0.001^**^ |

Values are mean ± SD (standard deviation), median (percentiles 25th-75th), or n (%). P value indicates comparison among distinct grades of blood pressure. P^**^ < 0.01. SBP: systolic blood pressure, DBP: diastolic blood pressure, HR: heart rate, TC: total cholesterol, HDL-c: high density lipoprotein-cholesterol, LDL-c: low density lipoprotein-cholesterol, FBG: fasting blood glucose, Cr: creatinine, ACR: ratio of albuminuria and creatinine, LA: left atrium, LV: left ventricle, LVPW: left ventricular posterior wall, IVS: interventricular septum, LVMI: left ventricular mass index, LVEF: left ventricular ejection fraction, Ang II: angiotensin II.

**References**

1. Nagpal V, Rai R, Place AT, Murphy SB, Verma SK, Ghosh AK, Vaughan DE. MiR-125b Is Critical for Fibroblast-to-Myofibroblast Transition and Cardiac Fibrosis. Circulation. 2016;133(3):291-301. doi:10.1161/circulationaha.115.018174.

2. Lang RM, Badano LP, Mor-Avi V, Afilalo J, Armstrong A, Ernande L, Flachskampf FA, Foster E, Goldstein SA, Kuznetsova T, Lancellotti P, Muraru D, Picard MH, Rietzschel ER, Rudski L, Spencer KT, Tsang W, Voigt JU. Recommendations for cardiac chamber quantification by echocardiography in adults: an update from the American Society of Echocardiography and the European Association of Cardiovascular Imaging. J Am Soc Echocardiogr. 2015 Jan;28(1):1-39 e14. doi:10.1016/j.echo.2014.10.003. Cited in: Pubmed; PMID 25559473.

3. Olson E, Pravenec M, Landa V, Koh-Tan HHC, Dominiczak AF, McBride MW, Graham D. Transgenic overexpression of glutathione S-transferase μ-type 1 reduces hypertension and oxidative stress in the stroke-prone spontaneously hypertensive rat. Journal of Hypertension. 2019;37(5):985-996. doi:10.1097/hjh.0000000000001960.

4. Di Mattia RA, Mariangelo JIE, Blanco PG, Jaquenod De Giusti C, Portiansky EL, Mundina-Weilenmann C, Aiello EA, Orlowski A. The activation of the G protein-coupled estrogen receptor (GPER) prevents and regresses cardiac hypertrophy. Life Sci. 2020 Feb 1;242:117211. doi:10.1016/j.lfs.2019.117211. Cited in: Pubmed; PMID 31891720.

5. Francois AA, Obasanjo-Blackshire K, Clark JE, Boguslavskyi A, Holt MR, Parker PJ, Marber MS, Heads RJ. Loss of Protein Kinase Novel 1 (PKN1) is associated with mild systolic and diastolic contractile dysfunction, increased phospholamban Thr17 phosphorylation, and exacerbated ischaemia-reperfusion injury. Cardiovasc Res. 2018 Jan 1;114(1):138-157. doi:10.1093/cvr/cvx206. Cited in: Pubmed; PMID 29045568.
